# Feasibility of an AI-Based Measure of the Hand Motions of Expert and Novice Surgeons

**DOI:** 10.1155/2018/9873273

**Published:** 2018-03-04

**Authors:** Munenori Uemura, Morimasa Tomikawa, Tiejun Miao, Ryota Souzaki, Satoshi Ieiri, Tomohiko Akahoshi, Alan K. Lefor, Makoto Hashizume

**Affiliations:** ^1^Department of Advanced Medical Initiatives, Faculty of Medical Sciences, Kyushu University, Fukuoka, Japan; ^2^TAOS Institute, Tokyo, Japan; ^3^Department of Advanced Medicine and Innovative Technology, Kyushu University Hospital, Fukuoka, Japan

## Abstract

This study investigated whether parameters derived from hand motions of expert and novice surgeons accurately and objectively reflect laparoscopic surgical skill levels using an artificial intelligence system consisting of a three-layer chaos neural network. Sixty-seven surgeons (23 experts and 44 novices) performed a laparoscopic skill assessment task while their hand motions were recorded using a magnetic tracking sensor. Eight parameters evaluated as measures of skill in a previous study were used as inputs to the neural network. Optimization of the neural network was achieved after seven trials with a training dataset of 38 surgeons, with a correct judgment ratio of 0.99. The neural network that prospectively worked with the remaining 29 surgeons had a correct judgment rate of 79% for distinguishing between expert and novice surgeons. In conclusion, our artificial intelligence system distinguished between expert and novice surgeons among surgeons with unknown skill levels.

## 1. Introduction

The relative importance of technical and nontechnical skills in surgical expertise is not well defined. Generally, the number of operations a surgeon has successfully performed is considered a valid indicator of surgical skill level; surgeons with more experience are considered “expert surgeons.” From a nontechnical point of view, expert surgeons are expected to be able to determine methods of overcoming intraoperative difficulties and to manage these difficulties independently. However, current methods of determining expertise based on years of experience tend to be subjective and not quantitative, and a valid definition of “expertise in laparoscopic surgery” is still under discussion [1]. The Japan Society for Endoscopic Surgery conducts the accreditation examination for laparoscopic surgery. Although there are some documented criteria, the judges subjectively evaluate nonedited video recordings of the examinees. Because the accreditation examination is a proven effective measure of clinical practice, judges' evaluations are considered objective and are reproducible, to some extent [2].

The ability to assess one's own performance critically in surgery is a valuable trait for surgeons throughout training and independent practice. This remains an underdeveloped skill in surgical training and receives little attention from surgical educators. For trainees, this skill allows identification of their surgical strengths and, more importantly, weaknesses, to build upon previous performance and to take the necessary remedial action. For surgeons in independent practice, introducing new surgical techniques necessitates focused self-assessment [3–7]. Our previous work focused on the hand motions of expert and novice surgeons. Kinematic data describing the motions of a surgeon's forceps during a skill assessment task were analyzed mathematically, revealing new insights about hand motion during laparoscopic surgery [8]. This method enables the surgical motions of expert and novice surgeons to be assigned objective, numerical values using analysis by chaotic time series mathematical theory. Accordingly, we developed a new concept in this study: an AI-based measure of the hand motions of expert and novice surgeons.

A neural network is an artificial intelligence (AI) system (constructed from artificial neurons) modeled after the way the human brain works and imitating how the brain's neurons are activated. Several computing cells work in parallel to produce a result, which is considered one of the ways that AI functions. Most neural networks process data that are weighted, and can tolerate unknown and varying input data. With labeled samples, neural networks executed by “normal computers” distinguish “normal computers” for logical algorithms. AI systems are most often used to estimate functions that depend on a large number of inputs and that are generally unknown. To estimate these functions, the AI system learns how its weight of each neural activity changes with time. Because the learning process is usually controlled by differences between the output and target values, the estimated function becomes gradually more precise in deriving the output values closer to the target values. When differences in hand motions between expert and novice surgeons are considered as the target values, the developed AI can estimate the function that computes the differences between the surgeons' hand motions.

The aim of the current study was to develop an AI system and to determine the feasibility of the system to distinguish the hand motions of expert and novice surgeons.

## 2. Materials and Methods

### 2.1. Study Participants

Participants in this study included 67 surgeons enrolled in a laparoscopic surgery training course held at the Kyushu University Training Center for Minimally Invasive Surgery [1, 8–12]. All participating surgeons performed the skill assessment task, which was described previously [1, 8, 9]. None of the participants were included in our previous study [8], and 38 were enrolled in study 1 (optimization study of the AI system) and the remaining 29 in study 2 (validation study of the AI system).

In study 1, 11 of the participants were expert surgeons, each of whom had performed more than 500 laparoscopic operations and who had completed the skill assessment task (expert group), and 27 were inexperienced surgeons, each of whom had performed fewer than 15 laparoscopic operations and who had not completed the skill assessment task (novice group).

In study 2, the expert group consisted of 12 of the participants and the novice group consisted of 17 participants, according to the criteria described above.

Participants voluntarily agreed to participate and gave informed consent to the staff of the Kyushu University Training Center for Minimally Invasive Surgery to publish their results.

### 2.2. Assessment Task and Objective Data Collection

The methodologies used for skill assessment and objective data collection were the same as in our previous study where they are described in detail [8]. Briefly, two identical needle holders were set into a box. A six-degree-of-freedom magnetic tracking sensor was mounted onto the tip of each needle holder. The box contained a stretched rubber sheet with a printed circle and eight pairs of dots. After tying two throws following the placement of the first suture at any pair of dots, the participant continuously sutured each pair of dots along the printed circle and ended with the final two throws tied to the tail of the first suture. The time allotted for the task was 7 minutes. The path of the tip of each needle holder was tracked using the magnetic tracking sensor, and the data were recorded. The data of the hand trajectories for the hand motions were used for all subsequent studies.

### 2.3. Input Factors for the AI System

In our previous study, we concluded that the flexibility of hand motion parameters could be analyzed using detrended fluctuation analysis and that the stability of the hand motion parameters could be analyzed using unstable periodic orbits analysis using time series data for both hand trajectories. Detrended fluctuation analyses were performed based on the four following factors:

- The paths of the center of gravity of both hands
- The relative paths of both hands
- The velocity of the center of gravity of both hands
- The relative velocity of both hands.

Unstable periodic orbits analyses were performed based on the following four factors:

- The second orbit of the paths of the center of gravity of both hands
- The third orbit of the paths of the center of gravity of both hands
- The second orbit of the velocity of the center of gravity of both hands
- The third orbit of the velocity of the center of gravity of both hands.

Details are in [8].

In the current study, we analyzed the factors listed above using the following AI system.

### 2.4. AI System

We constructed the AI system using the Neural Network Toolbox of MATLAB (The Mathworks Inc., Natick, MA, USA) to distinguish between experienced and novice surgeons. The AI system consists of a chaos neural network with three layers: an input layer, a hidden layer, and an output layer. The input layer consists of the eight previously identified input factors described earlier, the hidden layer consists of 30 neurons, and the output layer consists of two neurons as identifiers: 1 (expert) and 0 (novice).

**Study 1 (optimization study of the AI system).**

**Figure 1:**
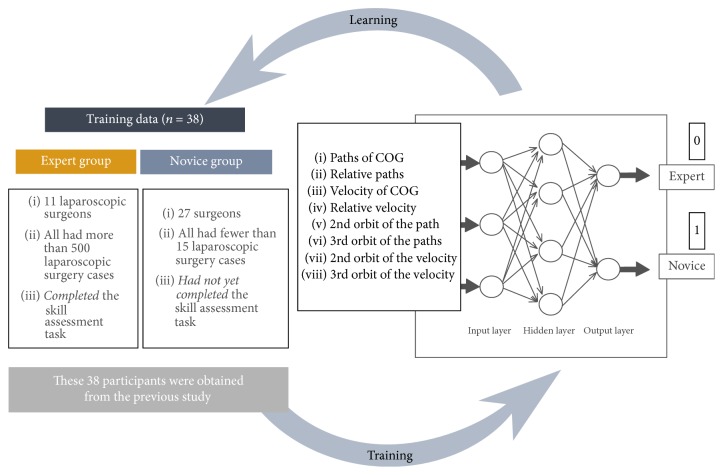
Optimization of the AI system. The AI system consists of a chaos neural network made of three layers: an input layer, a hidden layer, and an output layer. The input layer consists of eight previously identified input factors, the hidden layer consists of 30 neurons, and the output layer consists of two neurons as identifiers: 1 (expert) and 0 (novice). To optimize its ability to distinguish skills correctly, the neural network learned via machine learning from a training dataset consisting of parameters from 38 surgeons (11 experts and 27 novices).

To optimize its ability to correctly distinguish between experienced and novice surgeons, the neural network learned via machine learning from datasets consisting of input factors from 38 participants (expert group: 11; novice group: 27) (Figure 1). The backpropagation algorithm was employed as a learning strategy [13]. Learning by the system was repeated until the two groups of surgeons were correctly distinguished.

**Study 2 (validation study of the AI system).**

To validate the AI system, we entered 29 participants' (expert group: 12; novice group: 17) input factors into the system. The system was then tested for its ability to distinguish expert from novice surgeons, based on the eight identified factors. Correct classification of participants was the primary outcome.

## 3. Results

**Study 1 (optimization study of the AI system).**

**Figure 2:**
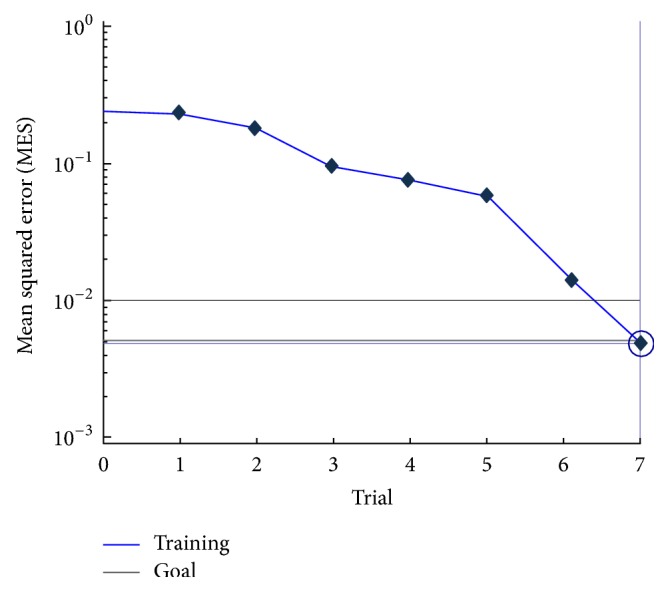
Optimization of the AI system during machine learning was completed by the seventh trial. The best training performance (mean squared error) was 0.0049 at trial 7.

Optimization of the AI system through machine learning using a training dataset consisting of parameters from 38 participants was completed by the seventh trial (Figure 2). The correct judgment ratio using the learning dataset was 0.99.

**Study 2 (validation study of the AI system).**

**Figure 3:**
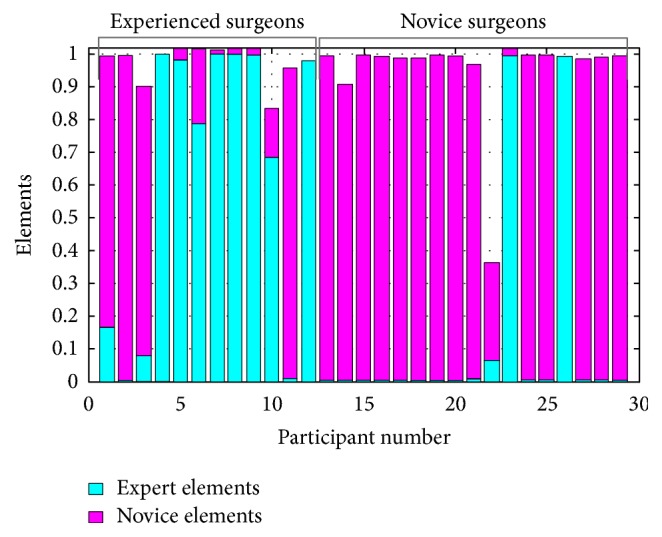
Ability of the AI system to distinguish between expert and novice surgeons. The blue elements of each bar are “expert elements,” and the pink elements are “novice elements” as computed by the neural network. Surgeons 1–12 were experts, and surgeons 13–29 were novices.

The AI system had a correct judgment rate of 79% for distinguishing between expert and novice surgeons.  Figure 3 shows the output of the neural network. The blue elements of each bar are “expert elements,” and the pink elements are “novice elements” as computed by the AI system. Surgeons 1–12 were actual experts and surgeons 13–29 were actual novices.

## 4. Discussion

The optimized AI system in this study correctly distinguished 79% of the test participants as expert or novice surgeons. There were no human interventions during classification, meaning that this result can be considered objective and quantitative. Although this system has a high classification accuracy ratio (79%), six errors were detected. There were four errors in classification (participants 1, 2, 3, and 11) in the expert group and two errors in classification (participants 23 and 26) in the novice group (Figure 3); four experts were judged to be novices and two novices were judged to be experts. We noted that the four participants in the expert group who were misclassified as novices had fewer years of experience (9.0 ± 4.3 years) than the group average (13.3 ± 5.0 years), but more than the average of the novice group (5.8 ± 5.0 years). The average number of years of experience of the two participants in the novice group who were misclassified was 2.5 ± 0.7 years. However, this does not explain why the two participants in the novice group were classified as experts in spite of their fewer years of experience. These data will be input as training data, and we plan to perform the AI learning cycle again. The trial and improvement method is the best way to construct a high-accuracy neural network [13, 14].

Our system made a number of misclassifications. Because we aimed to develop an AI system that distinguishes between experts and novices with no human interventions, we did not use expert surgeons to check whether the experts behaved like novices or whether the novices were just very good. Because the number of participants in study 1 was estimated at approximately 30, we decided to use 30 hidden neurons in the current study. The optimal number of hidden neurons according to the number of inputs is still controversial [15, 16]. The misclassifications may have been caused by our overfitting problem. Although fitting the number of neurons according to the number of inputs is controversial, the overfitting problem should be considered to improve our AI system.

The current study focused on surgeons' motor behaviors during surgery and found promising factors that may help define “surgical expertise” based on surgeons' hand motions. However, our methodology makes it difficult to fully understand surgical procedures from the results because we used only time series trajectories of hand motion and omitted procedural analysis. To define surgeons' expertise more concretely, a surgical processing model analysis needs to be added to our hand motion analysis in future studies.

**Figure 4:**
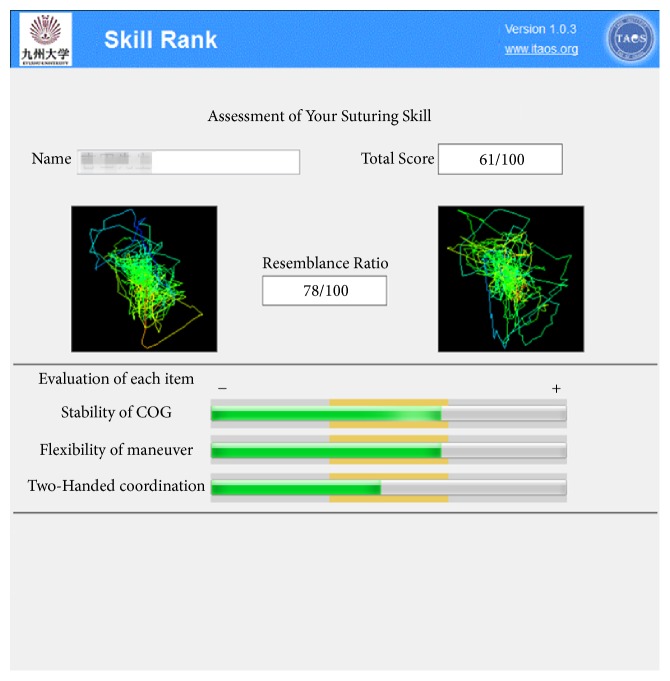
New concept for skill assessment using an AI-based measure of the hand motions of expert and novice groups. This system shows the results of the skill assessment quantitatively with a bar graph. The left side of the complex color line indicates features of novices' hand motions calculated by unstable periodic orbit analysis. The right side of the complex color line indicates features of average experts' hand motions calculated by unstable periodic orbit analysis. The resemblance ratio is the result of comparing results with those from the average from expert surgeons' data. The three evaluation items indicate the amount of each fluctuation calculated using detrended fluctuation analysis. The orange zone indicates the proper amount of fluctuation. Each zone was calculated by averaging the experts' results. Stability of COG, stability of the center of gravity of both hands; flexibility of maneuver, flexibility of maneuverability of both hands; two-handed coordination, coordination of both hands during surgery.

Based on this study, we developed a prototype AI model with a new concept (Figure 4). The program aims to clarify surgeons' skills in terms of what they have or have not mastered and their strengths and weaknesses, and the system provides feedback to surgeons to improve their skills, specifically and quantitatively.

In conclusion, using the factors identified in our previous study, our AI system was able to distinguish expert and novice surgeons among surgeons with unknown skill levels. In the future, we plan to further develop the AI-based measure to have higher accuracy to classify surgical skill and to develop a more useful surgical skill assessment system for training and education.

## Data Availability

The data that support the findings of this study are available on request from the corresponding author Munenori Uemura, because of ethical concerns.
